# Urine Eicosanoids in the Metabolic Abnormalities, Telmisartan, and HIV Infection (MATH) Trial

**DOI:** 10.1371/journal.pone.0170515

**Published:** 2017-01-24

**Authors:** Catherine N. Le, Todd Hulgan, Chi-Hong Tseng, Ginger L. Milne, Jordan E. Lake

**Affiliations:** 1 Vanderbilt University School of Medicine, Department of Medicine, Division of Infectious Disease, Nashville, Tennessee, United States of America; 2 University of California-Los Angeles, Department of Medicine, Division of Infectious Disease, Los Angeles, California, United States of America; Rush University, UNITED STATES

## Abstract

**Objectives:**

Arachidonic acid metabolites (eicosanoids) reflect oxidative stress and vascular health and have been associated with anthropometric measures and sex differences in cross-sectional analyses of HIV-infected (HIV+) persons. Telmisartan is an angiotensin receptor blocker and PPAR-γ agonist with potential anti-inflammatory and metabolic benefits. We assessed telmisartan’s effects on urine eicosanoids among HIV+ adults with central adiposity on suppressive antiretroviral therapy enrolled in a prospective clinical trial.

**Methods:**

Thirty-five HIV+ adults (15 women; 20 men) completed 24 weeks of open-label oral telmisartan 40mg daily. Lumbar computed tomography quantified visceral (VAT) and subcutaneous (SAT) abdominal adipose tissue. Urine F_2_-isoprostane (F_2_-IsoP), prostaglandin E_2_ (PGE-M), prostacyclin (PGI-M), and thromboxane B_2_ (TxB-M) were quantified at baseline and 24 weeks using gas/liquid chromatography-mass spectroscopy. Mann-Whitney-U tests compared sub-group differences; Spearman’s rho assessed correlations between clinical factors and eicosanoid levels.

**Results:**

Median PGE-M increased on telmisartan (p<0.01), with greater changes in men (+4.1 [p = 0.03] vs. +1.0 ng/mg cr in women; between-group p = 0.25) and participants losing >5% VAT (+3.7 ng/mg cr, p<0.01) and gaining >5% SAT (+1.7 ng/mg cr, p = 0.04). Median baseline F_2_-IsoP and TxB-M were slightly higher in women (both between-group p = 0.08) and did not change on telmisartan.

**Conclusions:**

Urine PGE-M increased with 24 weeks of telmisartan in virally suppressed, HIV+ adults with central adiposity. Associations with favorable fat redistribution suggest increased PGE-M may reflect a beneficial response.

## Introduction

With advances in antiretroviral therapy (ART), the management of comorbid conditions in persons with well-controlled HIV has become increasingly important. We now recognize the intricate interplay between traditional risk factors, ART-associated metabolic disturbances, and the inflammatory milieu created by chronic HIV infection that leads to cardiovascular disease, disorders of glucose-insulin homeostasis, and adipose tissue dysfunction.[1–8] Traditional clinical tools used to risk-stratify these diseases in HIV-uninfected persons may underestimate risk in patients with well-controlled HIV,[9] while biomarkers such as high-sensitivity C-reactive protein (hs-CRP) may be better surrogate markers for cardiovascular disease.[10,11]

Novel biomarkers may provide additional insight into the pathophysiology of inflammation-related disease in treated HIV infection. Eicosanoids are endogenous products of arachidonic acid metabolism associated with oxidative stress, inflammation, and endothelial dysfunction that can be measured in plasma, and their more chemically-stable metabolites can be reliably measured in urine.[12–14] Non-enzymatic oxidation of cell membrane phospholipids during cellular stress generates free radical oxidative species that are involved in the production of several biologically active eicosanoids, including F_2_-isoprostanes, which cause platelet activation, vasoconstriction, and endothelial damage. Membrane phospholipid release can also lead to COX-mediated metabolism of arachidonic acid generating prostaglandins, a family of products including thromboxanes, prostaglandin E, and prostacyclin. Their biologic properties and downstream effects are prolific, complex, and reviewed elsewhere.[15–17] Eicosanoids have been studied in a variety of disease processes including malignancy, autoimmunity, cardiovascular health, as well as HIV infection, and levels are known to be affected by environmental factors such as cigarette smoking.[18–28]

We have previously reported abnormal eicosanoids in HIV-infected persons, with differences noted between sexes and moderate associations with anthropometric measures.[29] Telmisartan is an angiotensin receptor blocker (ARB) and partial PPAR-γ agonist with potential metabolic and anti-inflammatory benefits,[30,31] including improvements in visceral adipose tissue (VAT) volume, fasting glucose and lipid profiles, and markers of oxidative stress and vascular inflammation in primarily HIV-uninfected populations.[32–35] The Metabolic Abnormalities, Telmisartan, and HIV Infection (MATH) trial was a pilot study designed to assess the effects of 24 weeks of telmisartan on visceral adiposity and metabolic parameters in HIV-infected participants with central adiposity on suppressive ART.[36] Given the reported associations between telmisartan therapy and improved oxidative stress, we sought to explore the effects of telmisartan on eicosanoid metabolites in stored urine samples from MATH participants.

## Methods

### Patient population and study design

Complete methods for the parent study have been published previously.[36] Briefly, 35 HIV-infected volunteers with evidence of central adiposity were enrolled in a single arm, open label pilot study between May and October 2010 at the UCLA Clinical AIDS Research and Education Center. Follow-up occurred through April 2011. The main inclusion criteria were: central adiposity defined as waist circumference >94 or 95 cm or waist-hip ratio >0.88 or 0.94 (for women and men, respectively)[37] and HIV infection on suppressive ART. Exclusion criteria germane to this analysis included current use of thiazolidinediones or ARBs; current use of nelfinavir or etravirine (due to concern for possible cyctochrome P-450 2C19 inhibition by telmisartan); untreated renal artery stenosis; unstable coronary artery disease, angina, or decompensated congestive heart failure; history of ARB intolerance; and need for ongoing potassium supplementation. Participants with hypertension were allowed to enroll if there had been no significant changes to their antihypertensive regimen in the 24 weeks prior to study entry and if the prescribing doctor approved the addition of telmisartan. Persons who were taking an angiotensin converting enzyme inhibitor, lipid-lowering therapy, or insulin-sensitizing medications were asked to not titrate these medications during the study period. Sexually active female participants were required to use contraception during and for at least four weeks after discontinuation of telmisartan. Menopausal status was self-reported and defined as >12 months since the last menstrual period in women with an intact reproductive system. All participants received oral telmisartan 40 mg daily with continued ART for 24 weeks. All participants provided written informed consent prior to the initiation of study procedures. This study was approved by the Institutional Review Board of the University of California, Los Angeles, and is registered at clinicaltrials.gov (NCT 01088295).

### Assessments

#### Urine eicosanoid measurements

Clean-catch urine samples were collected and stored at -70°C. Samples were shipped overnight on dry ice to the Vanderbilt University Eicosanoid Core Laboratory for analysis. Levels were quantified at weeks 0 and 24. Urinary 8-iso-PGF_2_αλπηα (F_2_-isoprostane P, or F_2_-IsoP), 11-dehydro-thromboxane B_2_ (TxB-M), and 2,3-dinor-6-keto-PGF_1_αλπηα (PGI-M) were measured using gas chromatography-negative-ion chemical ionization mass spectrometry employing stable isotope dilution methodology, as described elsewhere.[12,14,15,38] 9,15-dioxo-11-hydroxy-13,14-dihydro-2,3,4,5-tetranor-prostane-1,20-dioic acid (PGE-M) was measured by liquid chromatography-mass spectroscopy (LC-MS), as previously described.[13] Based on recent published data suggesting association with cardiovascular outcomes,[39] the ratio of thromboxane to prostacyclin urine metabolites was also calculated. Results for all urinary metabolites are presented as ng/mg urinary creatinine (cr).

#### Anthropometric measurements

Waist and hip circumferences were performed according to AIDS Clinical Trials Group standards[36] at weeks 0 and 24, and waist-hip ratios were calculated. Adipose tissue areas were measured by single-slice, L4-L5 computed tomography scan at weeks 0 and 24. Scans were performed at the UCLA Ronald Reagan Medical Center, and were standardized and read by a blinded reader at the Tufts University Body Composition Center.

#### Laboratory assessments

As described previously,[36] fasting (≥8 hours) glucose and lipoprotein profiles and HIV-1 RNA (assay sensitivity 50 copies/mL) were assessed at weeks 0 and 24.

### Statistical analysis

Statistical analyses were performed and figures were created using statistical software Graphpad Prism and SPS. Medians and interquartile ranges (IQR) are reported for continuous variables, and percentages for categorical data. Baseline characteristics between demographic subgroups were compared using the Mann-Whitney U-test for continuous variables. Spearman’s rho was used to evaluate correlations between continuous baseline and 24-week change variables. Primary outcomes for these analyses were the 24-week changes in urine eicosanoids. Median 24-week changes were compared using the Wilcoxon signed-rank test. The primary analysis was as-treated and excluded patients who did not remain on telmisartan and/or did not have 24-week follow-up data. Due to expected differences in eicosanoids and anthropometric characteristics by sex, additional pre-specified sex-stratified analyses were performed using Mann-Whitney U tests. Multivariate linear regression analyses were also performed, but the small sample size limited the number of covariates examined in each model. A p-value of ≤ 0.05 was deemed statistically significant. Due to the pilot nature of this study, all analyses were exploratory and no adjustment was made for multiple testing.

## Results

### Baseline characteristics

As previously reported, 47 persons were screened, 36 enrolled, and 35 completed the Week 24 primary endpoint.[36] Baseline demographic and clinical characteristics are described in Table 1. The median age was 49 years, body mass index (BMI) 31 kg/m^2^, and CD4^+^ T cell count 590 cells/μL. Thirty-three percent of women were current smokers compared to 55% of men (46% overall), and women were more likely to be black (53%) or Hispanic (47%) than men (20% black, 40% Hispanic). Forty percent of women (n = 6) were self-reported as post-menopausal. ART regimens at enrollment included 57% protease inhibitor (PI), 23% non-nucleoside reverse transcriptase inhibitor (NNRTI), and 23% raltegravir. Of NRTIs, the most commonly used were emtricitabine (66%) and tenofovir (74%). Median VAT at baseline was 179 cm^2^ and was higher in males (221 cm^2^) than females (113 cm^2^). Reflective of the median BMI of 31 kg/m^2^, median subcutaneous (SAT) and total (TAT) adipose tissue were also high (SAT 375 cm^2^, TAT 532 cm^2^) and similar across both sexes.

**Table 1:** Demographic and Clinical Baseline Characteristics^§^.

	Total (n = 35)		Women (n = 15)		Men (n = 20)	
Ethnicity*						
African-American	12 (34%)		8 (53%)		4 (20%)	
Hispanic	15 (43%)		7 (47%)		8 (40%)	
White	8 (23%)		0 (0%)		8 (40%)	
Age (years)	49 (44, 54)		50 (45, 54)		49 (46, 53)	
BMI (kg/m^2^)	31 (27, 35)		28 (26, 40)		31 (28, 33)	
Tobacco Use (Current)	16 (46%)		5 (33%)		11 (55%)	
CD4 count (cells/uL)	590 (455, 791)		736 (455, 1054)		565 (456, 660)	
PI	20 (57%)		8 (53%)		12 (60%)	
NNRTI	8 (23%)		2 (13%)		6 (30%)	
Integrase Inhibitor	8 (23%)		4 (27%)		4 (20%)	
NRTI Backbone	34 (97%)		15 (100%)		19 (95%)	
Abacavir	6 (17%)		3 (20%)		3 (15%)	
Lamivudine	8 (23%)		5 (33%)		3 (15%)	
Emtricitabine	23 (66%)		9 (60%)		14 (70%)	
Tenofovir	26 (74%)		11 (73%)		15 (75%)	
VAT (cm^2^)*	179 (113, 230)		113 (96, 159)		221 (176, 247)	
SAT (cm^2^)*	375 (250, 434)		426 (308, 813)		329 (237, 415)	
TAT (cm^2^)	532 (459, 615)		532 (433, 920)		530 (462, 600)	
VAT:SAT*	0.45 (0.26, 0.75)		0.24 (0.18, 0.37)		0.59 (0.43, 0.97)	
VAT:TAT*	0.31 (0.21 0.43)		0.19 (0.15, 0.28)		0.37 (0.30, 0.49)	
Waist Circumference (cm)	104 (99, 117)		103 (92, 128)		107 (100, 116)	
Hip Circumference (cm)	103 (97, 113)		109 (96, 138)		102 (97, 111)	
Waist-Hip Ratio*	1.01 (0.96, 1.06)		0.96 (0.93, 1.01)		1.05 (1.01, 1.09)	
SBP (mmHg)	130 (119, 144)		121 (117, 150)		131 (125, 143)	
DBP (mmHg)	80 (73, 84)		79 (69, 80)		80 (77, 86)	
Fasting Glucose (mg/dL)	95 (86, 99)		88 (81, 103)		96 (90, 98)	
Total Cholesterol (mg/dL)	184 (160, 211)		195 (154, 211)		181 (166, 212)	
Triglycerides (mg/dL)*	115 (94, 189)		112 (86, 124)		160 (101, 230)	
LDL (mg/dL)	102 (81, 128)		106 (81, 140)		100 (79, 125)	
HDL (mg/dL)*	45 (37, 52)		52 (40, 57)		41 (35, 46)	
Diabetes^†^	2 (9%)		1 (7%)		2 (10%)	
Hypertension^†^	12 (34%)		3 (20%)		9 (45%)	
Hyperlipidemia^†^	16 (46%)		4 (27%)		12 (60%)	
Hepatitis B	2 (9%)		0 (0%)		2 (10%)	
Hepatitis C	3 (9%)		2 (13%)		1 (5%)	
Menopausal	N/A		6 (40%)		N/A	
Urinary eicosanoids (ng/mg cr)	Median (IQR)	Mean (SD)	Median (IQR)	Mean (SD)	Median (IQR)	Mean (SD)
F_2_-IsoP	1.19 (0.93, 2.12)	1.75 (1.3)	2.18 (0.91, 3.22)	2.26 (1.49)	1.11 (0.94, 1.32)	1.32 (0.96)
PGE-M	8.07 (4.45, 11.5)	9.11 (6.76)	6.32 (4.82, 10.7)	7.81 (4.46)	9.0 (8.91, 11.7)	10.1 (8.04)
PGI-M	0.09 (0.07, 0.12)	0.10 (0.06)	0.12 (0.07, 0.14)	0.12 (0.06)	0.08 (0.06, 0.10)	0.09 (0.05)
TxB-M	0.21 (0.11, 0.32)	0.24 (0.16)	0.32 (0.14, 0.46)	0.31 (0.20)	0.18 (0.12, 0.24)	0.19 (0.09)

^§^ Percent or median with interquartile range

* Between-group p ≤ 0.05

^†^ Defined as self-reported diagnosis or on medication at baseline.

BMI = body mass index; PI = protease inhibitor; NNRTI = non-nucleoside reverse transcriptase inhibitor; NRTI = nucleoside reverse transcriptase inhibitor; VAT = visceral adipose tissue; SAT = subcutaneous adipose tissue; TAT = total adipose tissue; SBP = systolic blood pressure; DBP = diastolic blood pressure; LDL = low-density lipoprotein cholesterol; HDL = high-density lipoprotein cholesterol; PGE-M = 11-hydroxy-9,15-dioxo-2,3,4,5-tetranor-prostane-1,20-dioic acid; F_2_-IsoP = F2-isoprostane P or 8-iso-PGI2-alpha; PGI-M = 2,3-dinor-6-keto-PGF1; TxB-M = thromboxane B_2_ or 11-dehydro-thromboxane B2

### Baseline urinary eicosanoids (Table 1 and Fig 1)

Baseline median (IQR) urinary PGE-M, F_2_-IsoP, PGI-M, and TxB-M were 8.07 (4.09, 11.55), 1.19 (0.97, 2.25), 0.09 (0.07, 0.23), and 0.21 (0.06, 0.13) ng/mg cr, respectively. F_2_-IsoP and TxB-M were both higher in females (2.18 ng/mg cr [0.79, 3.71] and 0.32 ng/mg cr [0.12, 0.49]) than males and approached statistical significance (both with between-group p = 0.08). PGE-M was higher in post-menopausal (10.75 ng/mg cr [10.05, 11.81]) compared to pre-menopausal women (6.32 ng/mg cr [3.03, 6.32], p = 0.04). TxB-M -to-PGI-M ratio did not differ significantly between subgroups (data not shown). PGE-M and TxB-M levels correlated with waist-hip ratio in females (rho = 0.39, p = 0.04; rho = 0.59, p = 0.02) with a similar relationship for PGE-M observed overall (rho = 0.30, p = 0.08).

**Fig 1:**
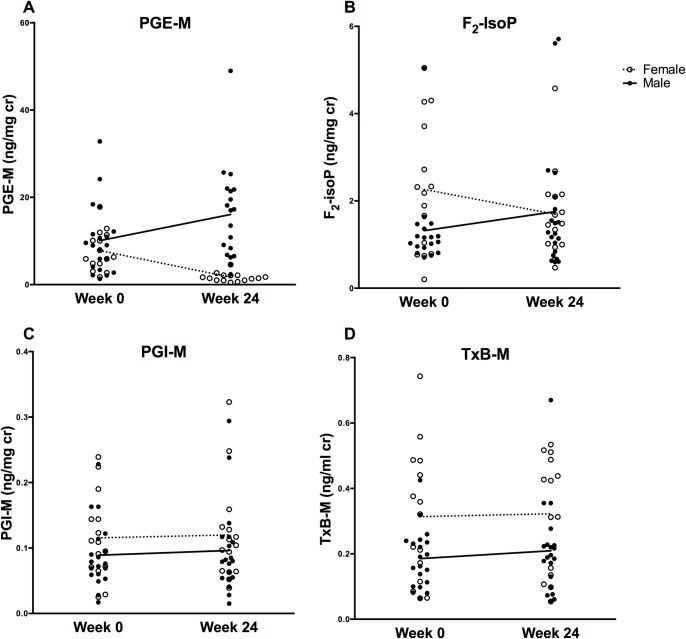
Urine eicosanoid levels at baseline and 24 weeks of telmisartan. Panel A: Scatter plot of baseline and 24-week urine PGE-M levels (ng/mg cr). Solid lines represent median change in male participants, dotted lines represent median changes in females. Mann-Whitney U p = 0.03 (males) and p = 0.12 (females). Panel B: Scatter plot of baseline and 24-week urine F_2_-IsoP levels (ng/mg cr). Mann-Whitney U p = 0.06 for males, p = 0.19 for females. Panel C: Scatter plot of baseline and 24-week urine PGI-M levels (ng/mg cr). Mann-Whitney U p = 0.71 for males, p = 0.76 for females. Panel D: Scatter plot of baseline and 24-week urine TxB-M levels (ng/mg cr). Mann-Whitney U p = 0.84 for males, p = 0.95 for females.

### Changes in urinary eicosanoids after 24 weeks of telmisartan (Table 2, Fig 2)

After 24 weeks, there were no statistically significant changes in median PGI-M or TxB-M (+0.0 ng/mg cr [-0.03, 0.04], p = 0.78, and +0.01 ng/mg cr [-0.06, 0.07], p = 0.71, respectively). However, PGE-M increased significantly (1.58 ng/mg cr [-0.12, 5.94], p = 0.008). This change was greater in men (+4.08 ng/mg cr [-0.43, 13.1], p = 0.03) than women (+1.03 ng/mg cr [0.06, 3.46], p = 0.12, between-group p = 0.03). Overall, F_2_-IsoP did not significantly change overall or by sex (+0.14 [-0.47, 0.45], p = 0.78; +0.35 ng/mg cr [-0.09, 0.57], p = 0.06 in males; 0.14 ng/mg cr [-0.75, 0.25], p = 0.19 in females), although sub-group analysis revealed a significant difference between changes in males compared to females (between-group p = 0.03). Levels of TxB-M at 24 weeks were slightly lower in males (0.19 ng/mg cr [0.12, 0.23]) than females (0.37 ng/mg cr [0.14, 0.48]), but the change was not significantly different between groups (p = 0.09). In a sub-group analysis of pre-menopausal versus post-menopausal women, no significant differences in median eicosanoid changes were seen, and both levels at week 24 and change values were similar.

**Fig 2:**
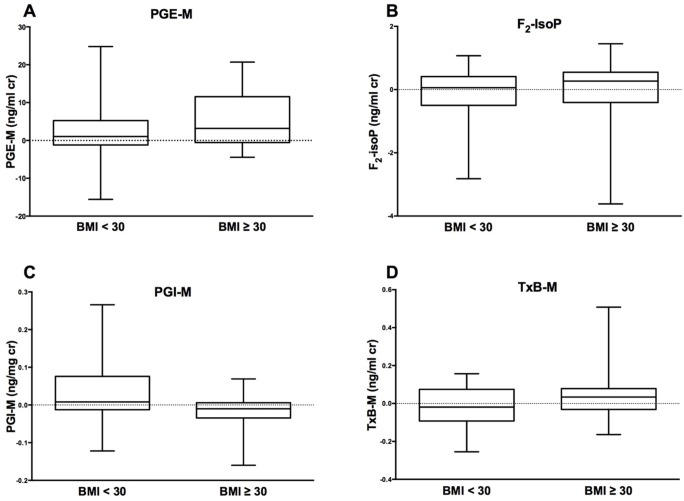
Change in urine eicosanoid levels after 24 weeks of telmisartan by BMI. Panel A: Box and whisker plot of urine PGE-M levels (ng/mg cr). Maximum and medium values are denoted by whiskers and solid line, respectively. Mann-Whitney U p = 0.40. Panel B: Box and whisker plot of urine F_2_-IsoP levels (ng/mg cr). Mann-Whitney U p = 0.52. Panel C: Box and whisker plot of urine PGI-M levels (ng/mg cr). Mann-Whitney U p = 0.04. Panel D: Box and whisker plot of urine TxB-M levels (ng/mg cr). Mann-Whitney U p = 0.31.

**Table 2:** Absolute and 24-week changes in urinary eicosanoid levels by study arm (median with interquartile range).

	Total (n = 35)	Within-group p	Women (n = 15)	Within-group p	Men (n = 20)	Within-group p	Between-group p
F_2_-IsoP							
Week 0	1.19 (0.97, 2.25)		2.18 (0.79, 3.71)		1.11 (0.90, 1.39)		0.080
Week 24	1.45 (0.94, 2.10)		1.48 (1.01, 2.15)		1.27 (0.77, 2.01)		0.629
Week 24 change	0.14 (-0.47, 0.47)	0.782	-0.15 (-0.88, 0.27)	0.188	0.35 (-0.12, 0.62)	0.060	0.027
PGE-M							
Week 0	8.07 (4.09, 11.55)		6.32 (4.8, 11.38)		9.0 (3.54, 12.01)		0.633
Week 24	10.5 (6.60, 17.48)		9.13 (6.30, 12.36)		17.02 (6.82, 21.78)		0.043
Week 24 change	1.48 (-0.55, 6.22)	0.008	1.03 (-0.29, 3.61)	0.107	4.08 (-1.32, 14.76)	0.029	0.245
PGI-M							
Week 0	0.09 (0.06, 0.13)		0.12 (0.07, 0.14)		0.08 (0.06, 0.11)		0.160
Week 24	0.09 (0.06, 0.12)		0.10 (0.06, 0.13)		0.08 (0.05, 0.12)		0.238
Week 24 change	-0.01 (-0.03, 0.07)	0.973	-0.01 (-0.03, 0.07)	0.762	-0.01 (-0.03, 0.07)	0.687	0.795
TxB-M							
Week 0	0.21 (0.11, 0.32)		0.32 (0.12, 0.49)		0.18 (0.10, 0.24)		0.083
Week 24	0.22 (0.20, 0.37)		0.37 (0.13, 0.49)		0.19 (0.12, 0.23)		0.090
Week 24 change	0.01 (-0.06, 0.07)	0.774	0.03 (-0.06, 0.07)	0.952	-0.01 (-0.9, 0.12)	0.870	0.796

PGE-M = 11-hydroxy-9,15-dioxo-2,3,4,5-tetranor-prostane-1,20-dioic acid; F_2_-IsoP = F2-isoprostane P or 8-iso-PGI2-alpha; PGI-M = 2,3-dinor-6-keto-PGF1; TxB-M = thromboxane B_2_ or 11-dehydro-thromboxane B2

### Correlations between change in urinary eicosanoids and clinical parameters

#### Correlations with anthropometric data

Overall, there were no significant correlations between median changes in PGE-M, F_2_-IsoP, PGI-M, and TxB-M and median percent changes in BMI, SAT, VAT, TAT, VAT-SAT ratio, or VAT-TAT ratio. Median changes in TxB-M/PGI-M ratio correlated with decreasing VAT (rho = -0.41, p = 0.02), VAT-SAT ratio (rho = -0.42, p = 0.02), and VAT-TAT ratio (rho = -0.37, p = 0.04). Males saw a trend between changes in PGE-M levels and hip circumference (rho = 0.39, p = 0.10), but no significant correlations were observed in women. Participants with baseline BMI ≥30 kg/m^2^ had a decrease in urine PGI-M levels after 24 weeks (-0.01 ng/mg cr [-0.03, 0.01], p = 0.17) compared to patients with BMI <30 kg/m^2^ (+0.01 ng/mg cr [-0.01, 0.08], p = 0.22; between-group p = 0.04) (Fig 2).

Statistically significant PGE-M increases were observed in participants losing >5% VAT (+3.7 ng/mg cr [0.43, 6.32], p = 0.002), but not among those gaining >5% VAT (+1.03 ng/mg cr [-4.44, 9.48], p = 0.57; between-group p = 0.37). The threshold of 5% was chosen given the association with increased risk of metabolic syndrome with VAT increases of ≥ 5%.[40] Participants gaining >5% SAT (+1.7 ng/mg cr [0.67, 10.60], p = 0.04) had significant increases in PGE-M, but those who lost >5% SAT did not (+1.03 ng/mg cr [-1.43, 4.78], p = 0.10; between-group p = 0.35). There were no statistically significant relationships between F_2_-IsoP, PGI-M, and TxB-M and VAT or SAT gain/loss.

#### Correlations with changes in other metabolic parameters

Overall, median changes in urine PGE-M and F_2_-IsoP positively correlated with changes in systolic blood pressure (rho = 0.48, p = 0.003 and rho = 0.35, p = 0.04 respectively). TxB-M/PGI-M ratio was inversely correlated with high-density lipoprotein (HDL) cholesterol levels (rho = -0.38, p = 0.03). Sub-group analyses by sex revealed heterogenous results with no notable correlations observed in females. Men saw a relationship with low-density lipoprotein (LDL) cholesterol levels and both F_2_-isoP (rho = 0.53, p = 0.02) and TxB-M (rho = 0.46, p = 0.04).

## Discussion

This study prospectively assessed the effects of telmisartan therapy on urine eicosanoid measurements in treated HIV infection; to our knowledge the first to do so. In this group of HIV-infected adults with central adiposity on suppressive ART, 24 weeks of telmisartan resulted in a significant increase in PGE-M but not F_2_-IsoP, PGI-M, or TxB-M. Subgroup analysis revealed baseline differences by sex, with females having higher F_2_-IsoP and lower PGE-M. When adjusting for race in multivariate regression models, the relationship between F_2_-IsoP and sex remained robust while PGE-M was not associated with sex, with or without adjusting for race.

Sex-specific differences in eicosanoids have been studied previously, showing that females had higher urinary F_2_-IsoP and TxB-M and lower PGI-M levels compared to men.[41] This profile is similar to smokers and persons with higher BMI, despite having a low Framingham risk score. Several studies in HIV-uninfected persons demonstrated similar trends,[42–44] but contrasting results exist. Our subgroup analysis revealed higher median levels of PGE-M in post-menopausal women (N = 6, 40%) that were similar to levels in men without significant changes in other eicosanoids after 24 weeks (data not shown). These findings are similar to a previous study[45]. While these results suggest higher levels of oxidant stress in females, male sex is a known risk factor for several diseases including cardiovascular disease and various malignancies, and post-menopausal women are also at higher risk for cardiovascular disease.[3,46] The small sample size of our study limits our ability to make substantial statements about relationships between the other urine eicosanoids and menopausal status, but these findings are part of a growing body of data suggesting a relationship between oxidative stress, sex hormones, and complications of HIV infection and treatment that warrants further investigation.

Sex differences in urine eicosanoid metabolites were also observed after 24 weeks of telmisartan therapy and were associated with favorable changes in AT redistribution including reductions in VAT by CT scan. There was an overall increase in PGE-M and F_2_-IsoP, with the greatest increases among men and persons losing VAT and gaining SAT. In addition, because the ratio of thromboxane to prostacyclins has been shown to correlate with increased cardiovascular disease,[39,47] we examined the relationship between the thromboxane-prostacyclin ratio and anthropometric parameters and found significant relationships between increasing thromboxane-prostacyclin ratio and favorable changes in adipose tissue distribution. A previous study measuring urine eicosanoids and central adiposity in women on suppressive ART before and after switching from a PI- or NNRTI-based regimen to a raltegravir-based regimen also noted a marginal positive correlation between changes in VAT and PGI-M and PGE-M.[29,48] Additionally, a study of the effects of the PPAR-γ agonist rosiglitazone on HIV-infected patients with lipodystrophy demonstrated an increase in *plasma* F_2_-IsoP levels associated with increased limb fat as well as changes in PGI-M that were BMI dependent.[49] The influence of adiposity on oxidative stress and eicosanoids has been echoed in studies of HIV-uninfected populations as well, where one study demonstrated a BMI-dependent association between urine F_2_-IsoP and risk of breast cancer, where F_2_-IsoP had a protective effect in participants with BMI <23 kg/m^2^ but was simultaneously associated with a nearly 10-fold increased risk of developing breast cancer in patients with BMI ≥29 kg/m^2^.[19]

Though the pilot nature of this study prohibits us from making definitive statements regarding the relationship between urine eicosanoids, telmisartan, and adiposity, the intriguing trends and preliminary associations we observed do suggest that some aspect of oxidative stress associated with central adiposity was altered by telmisartan. The biological precursor of PGE-M, PGE_2_, is a pleiotropic immune-modulatory molecule with both deleterious and beneficial effects on oxidative stress, having been shown *in vitro* to play a role in reducing cell-to-cell infectivity of HIV-1,[50] while also postulated to promote immune dysfunction and inefficient immune surveillance in HIV-infected persons.[51] Urinary PGE-M has been shown to correlate with plasma HIV-1 RNA and cervical cyclooxygenase-2 levels in Haitian women[52] and is increased in various malignancies in HIV-uninfected persons.[24,53–55] Whether PGE_2_ serves as a mediator or biomarker of inflammatory processes is not entirely clear. Similarly, F_2_-IsoP is more typically associated with increased inflammation and oxidative stress and has been extensively correlated with cardiovascular disease, obesity, malignancy, insulin resistance, smoking, and liver disease.[19,20,22,27,56] While it has been shown to be a sensitive and independent marker of cardiovascular disease,[21] one study demonstrated an inverse correlation between F_2_-IsoP levels and HIV-1 RNA levels.[26] In summary, descriptions of the specific actions of eicosanoid metabolites are controversial and sometimes contradictory, and the fact that both markers increased in our study despite favorable changes in adipose tissue distribution only further suggests that the behavior of these agents *in vivo* remains unclear and requires further evaluation.

Of course, given the small sample size of this pilot study, multivariate analyses were limited and likely led to underpowered analyses; thus the conclusions presented here are provocative but exploratory. Furthermore, as previously discussed in the parent study,[36] several confounding factors may have contributed to and influenced levels of oxidative stress and inflammation. While analyses were believed to be as-treated, telmisartan adherence was not formally assessed with pill counts or blood drug levels. Additionally, menopausal status was self-reported and we did not exclude current smokers; however, a secondary analysis excluding smokers did not significantly affect results. Participants using non-steroidal anti-inflammatory drugs or aspirin were not excluded from the study, nor were persons with chronic inflammatory comorbidities such as diabetes mellitus, hyperlipidemia, and hepatitis C virus co-infection. The median BMI of the study population was >30 kg/m^2^, which may have contributed to baseline inflammation and limited the efficacy of telmisartan (the metabolic effects of which have mainly been described in non-obese patients).[31,33] The designated follow up period of 24 weeks may have been insufficient to see substantial changes in urine eicosanoids or metabolic parameters in response to telmisartan. The parent trial observed statistically significant declines in SAT and TAT but was underpowered to see statistically significant results with similar changes in VAT. In this analysis, changes in urinary eicosanoids correlated with VAT loss and SAT gain, suggesting that the alterations in oxidant stress pathways may be related to the response of fat depots to telmisartan therapy. Finally, this was a single arm, open label study, and the lack of a control group prevents us from making comparisons to telmisartan-treated, HIV-uninfected persons or HIV-infected persons with poor virologic control or remaining on stable ART alone. Larger studies are needed to explore sex-specific differences and differential responses of urine eicosanoid metabolites to ARBs and other therapies.

## Conclusions

Urine PGE-M increased after 24 weeks of telmisartan therapy in HIV-infected adults on suppressive ART with central adiposity, with men and persons losing VAT and gaining SAT having the greatest increases. Associations with favorable adipose tissue redistribution suggest that increased PGE-M reflects a beneficial response. These pilot data suggest the utility of urine eicosanoids as potential biological mediators and/or biomarkers for inclusion in future studies in HIV-infected populations.

## Supporting Information

S1 CONSORT Checklist CONSORT Checklist. (TIF)

S1 File MATH protocol. Original protocol for the Metabolic Abnormalities, Telmisartan, and HIV infection (MATH) trial.
(PDF)
