# Supplementary figures and images for: Urine Eicosanoids in the Metabolic Abnormalities, Telmisartan, and HIV Infection (MATH) Trial
Source: PLoS One. 2017 Jan 24;12(1):e0170515. doi: 10.1371/journal.pone.0170515 (PMC5261803; doi:10.1371/journal.pone.0170515)

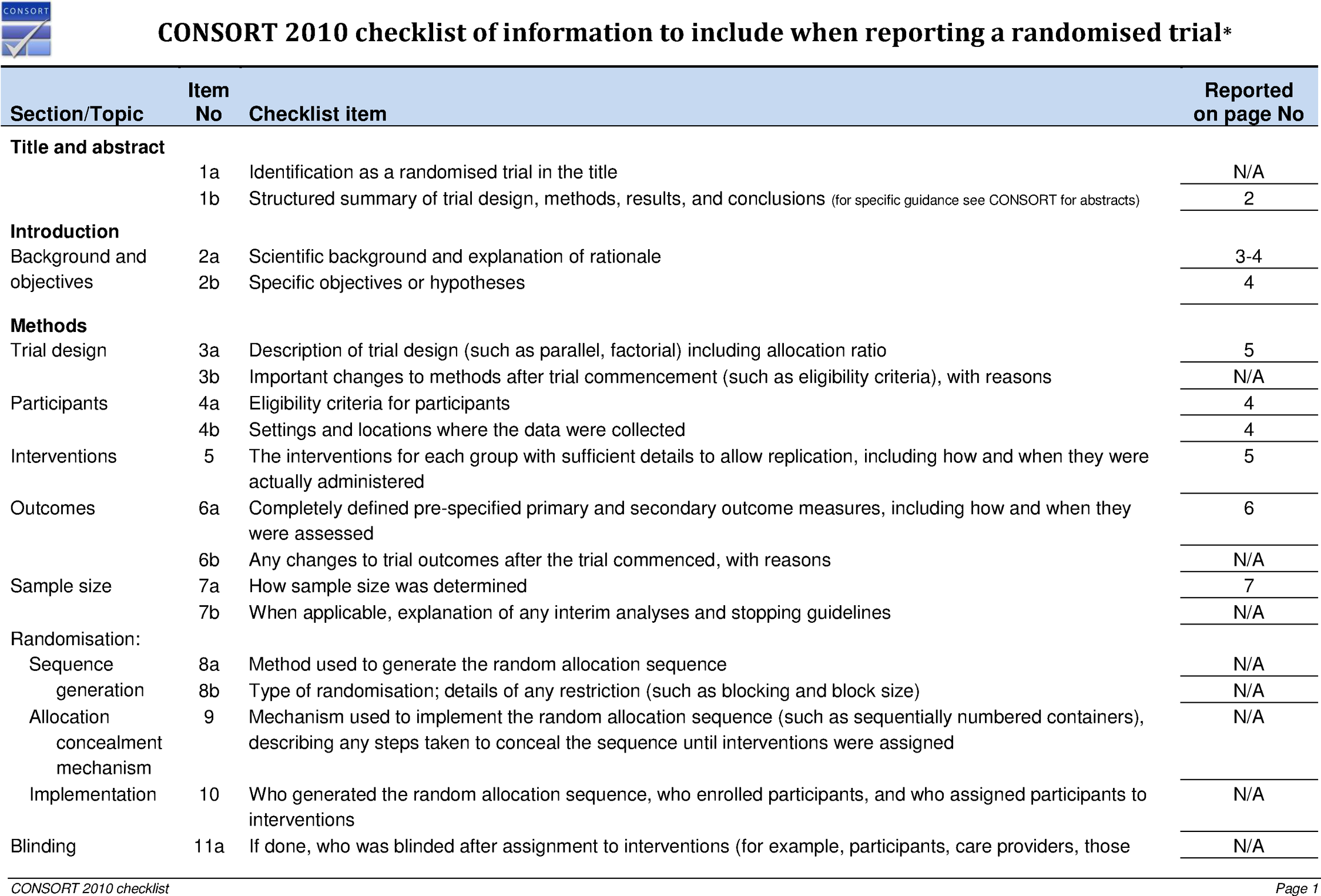

Supplement: S1 CONSORT Checklist — (TIF) [file pone.0170515.s001.tif]
